# Future Directions in Alcoholism Research: Genomics and Gene Transfer

**Published:** 2000

**Authors:** P. J. Brooks, Robert H. Lipsky

**Affiliations:** P.J. Brooks, Ph.D., is a Senior Staff Fellow and Robert H. Lipsky, Ph.D., is a research scientist at the Laboratory of Neurogenetics, National Institute on Alcohol Abuse and Alcoholism, Bethesda, Maryland

**Keywords:** gene expression, protein synthesis, genome, virus, mRNA, hippocampus, ventral tegmental area, animal model

## Abstract

Alcohol affects the process by which genes direct the synthesis of proteins (i.e., expression). Therefore, patterns of gene expression in the presence of alcohol can help scientists identify the specific molecular sites of alcohol’s actions within the brain. New technologies can detect and quantify changes in the expression of thousands of genes simultaneously by scanning microscopic gene arrays applied to glass or silicon chips an inch or so square. However, genes whose activity is altered in the presence of alcohol may either be contributing to alcoholism development or may be reacting to alcohol’s presence. This question can be researched by observing the effects of manipulating the level of specific gene products. One way to accomplish this end is by means of viruses that have been engineered to express a specific gene in infected cells. This technique has been applied successfully in studying addictive behaviors. It is suggested that patterns of gene expression may become a diagnostic tool, with different disease states being characterized by distinct expression profiles.

Polonged or repeated exposure to alcohol can lead to long-term changes in the function of nerve cells (i.e., neurons) within the brain. Researchers believe that these changes underlie certain manifestations of addictive behavior, such as tolerance, withdrawal, and the persistent craving for alcohol that appears to provoke relapse after prolonged abstinence. The molecular mechanisms underlying these long-term neurological changes largely involve specific brain proteins that play various roles in communication among neurons.

Information encoded in a cell’s genetic material directs the synthesis of a given protein. Thus, in whole organisms, the coordinated control of genes determines an individual’s basic structure. Minor variations among genes account for the normal range of inherited differences between individuals in a population. Conversely, major genetic variation may underlie an individual’s vulnerability to disease. At its most basic level, a dormant gene may become active in response to chemical messengers that signal a cell’s increased need for the gene’s particular protein product. The genetic information contained in the DNA is transcribed in the cell’s nucleus into a form that can be interpreted by the protein-synthesizing components of the cell called messenger RNA (mRNA). The process by which a gene changes its activity in directing the synthesis of its specific mRNA and the resulting protein is called expression.

Research indicates that alcohol affects gene expression (Bachtell et al. 1999). Furthermore, the pattern of gene expression in the presence of alcohol provides evidence for scientists to deduce the specific molecular sites of alcohol’s action within the brain (Miles 1995). This article focuses on two new approaches for analyzing gene expression that show potential for use in aspects of alcoholism research.

## Gene Expression

### Differential Expression

The differential expression approach detects and quantifies alterations in gene expression by indirectly measuring mRNA levels. Using this approach, Chen and colleagues (1997) studied differential expression in male rats after long-term (14-day) administration of alcohol. The investigators determined the total RNA content of specific brain regions. One significant difference detected in the alcohol-exposed rats was a striking elevation of a specific mRNA in the hippocampus that lasted up to 48 hours after withdrawal from alcohol (Chen et al. 1997). The hippocampus is involved in learning and memory and may play a role in alcohol-induced memory blackouts as well as seizures that often accompany the acute withdrawal syndrome following cessation of heavy drinking. The specific mRNA was determined to play a role in the synthesis of an enzyme crucial to energy metabolism in mitochondria. Mitochondria are structures within cells where most of the cell’s energy is produced. Based on these considerations, the results of the experiment of Chen and colleagues supports the idea that alcohol exposure causes defects in mitochondria that may also play a role in such health consequences as alcohol-induced liver disease.

In a comparison study of human brain tissue obtained post mortem from alcoholics and nonalcoholics, Fan and colleagues (1999) measured levels of different types of mRNA obtained from different brain regions. Levels of a specific mRNA were higher in the nucleus accumbens of alcoholic brains compared with nonalcoholic brains. This differentially expressed mRNA is known to play a role in the final stages of mitochondrial protein synthesis. The nucleus accumbens is a center of motivation and stress response and is implicated in the development of alcoholism. Taken together, these results are consistent with the possibility that alcohol-induced activation of energy metabolism in the nucleus accumbens plays a role in alcoholism development.

### Automated Gene Expression Analysis

New technologies offer quantitative and simultaneous monitoring of the expression of thousands of genes. The genes to be studied are applied in microscopic liquid droplets to a chemically treated glass slide, silicon chip, or similar surface often as small as a postage stamp. The spots eventually dry, forming a so-called microarray or biochip. An automated operating system scans the microarray and can calculate the relative expression levels of up to 10,000 selected genes simultaneously (Ermolaeva et al. 1998). Some systems can scan more than 100 different microarrays at once.

This approach can identify and characterize genes not previously suspected to be alcohol related. As more alcoholism researchers begin to employ this procedure, it may become possible to identify virtually every gene (and its protein product) that plays a significant role in alcohol-related behavior.

Mass-produced, “pre-spotted” microarrays are available commercially and include human, plant, animal, and pathogen genomes. Specific arrays are also offered, including human and rat biochips designed specifically for the study of neurobiology, each with approximately 1,000 genes that code for brain proteins likely to be involved in psychiatric and addictive disorders.

Automated gene expression profiling is complemented by recent advances in gene mapping (see the article in this issue by Grisel, pp. 169–174). The Human Genome Project has essentially completed the mapping of all our 80,000 to 100,000 genes (van Ommen et al. 1999). Complete genome sequences are also available for more than 30 non-human organisms, including many species that are commonly used in alcoholism research (Adams et al. 2000); sequencing of the laboratory mouse genome is in progress. Therefore, DNA sequences affixed to microarray chips can be compared against collections of human reference sequences, many publicly available through the Internet (Ermolaeva et al. 1998; Lash et al 2000). Gene expression techniques can also be applied to the sequencing of DNA.

## Viral Vectors

### How Human Genes Can Hitch Rides on Viruses

The differential gene expression approaches described in the first part of this article, as well as other genetic approaches, can provide a set of “candidate” genes that are differentially expressed in the alcoholic brain. However, these genes may either be involved in the pathogenesis of alcoholism or be the consequence of the alcoholic state. One way to distinguish between these possibilities is to manipulate the levels of the specific gene products and assess the effect of such manipulation on behavior such as alcohol consumption.

Commonly used approaches to manipulating genes in animals at present are the production of knockout and transgenic mice (see the article in this issue by Bowers, pp. 175–184). Although such approaches are quite effective, they are very time consuming, requiring the breeding of several generations of mice to achieve the desired genotypes. This limitation may become more severe as the number of candidate genes to be studied increases dramatically due to the increased use of array technologies. A more immediate approach to manipulating the level and type of specific gene products in cells is to use viral vectors. These are viruses or viral derivatives that have been engineered to express a gene of interest in infected cells. Using this approach, the level of a specific gene product in a brain region of interest can be increased simply by injecting a viral vector designed to express the gene into a specific brain region. One can then assess the effect of the manipulation of behavior or other parameters of relevance to alcoholism. In theory, it is also possible to reduce the levels of a gene product by injecting a viral vector that inhibits the messenger RNA encoded by the endogenous gene.

At present, there are several different viral vector systems in common use (Kaplitt and Loewy 1995), although they have not been widely used in alcoholism research. Herpes (Simonato et al. 1999) and adenoviral (Horellou et al. 1997) vectors are the most commonly used for gene transfer in the nervous system. The viral vectors can be genetically engineered to activate a target gene in a selected site at a desired time (Brooks et al. 1997). Viral vectors can be applied at any stage of the animal’s development, as opposed to knockout and transgenic mice in which the genetic alteration is performed during the embryonic stage. The site specificity of viral vectors within the brain can be an advantage or disadvantage depending on the goals of the experiment. A potential disadvantage is localized immune reactions at the site of injection (Wood et al. 1994, 1996).

Pathogenic viral vectors can be genetically modified to reduce the risk of their causing disease in the host organism. For example, brain researchers studying poliovirus vectors for targeted gene therapy (Bledsoe et al. 2000) have constructed poliovirus genomes that express specific human proteins in place of certain viral proteins. This system generates no active poliovirus, but produces transient, high levels of the inserted human genes.

Gene expression within the brain mediated by a viral vector shuts off after a period of days to months (McCown et al. 1996). Improvements in vector design are minimizing this issue (e.g., Kaplitt et al. 1994*a*,*b*; Haberman et al. 1998), although for some behavioral experiments, the reversibility of gene expression from viral vectors could be an advantage. For example, if a behavioral effect is seen while vector-derived gene expression is high, then disappears after the gene is shut off, this would be additional evidence for a role of the gene product in the behavior under study.

### Viral Vectors in Alcohol Research

Whereas the use of viral vectors for gene transfer in alcohol research is just beginning, this approach has been used successfully in studying other addictive behaviors. Carlezon and colleagues (1997) have succeeded in manipulating sensitivity of morphine reward by using a herpes simplex virus to transfer a glutamate receptor into cells of the ventral tegmental area in rats. Glutamate receptors are proteins involved in communication between nerve cells and are known to mediate some of alcohol’s effects (e.g., sedation, intoxication); the ventral tegmental area is one of a group of brain regions linked together that play a role in the rewarding effects of alcohol and other drugs. The same researchers utilized this approach to demonstrate the role of a different neuronal protein in modulating the rewarding aspects of cocaine (Carlezon et al. 1998). Notably, in these studies behavioral effects were observed despite the fact that only a small group of neurons within the target areas was affected. The successful use of the gene transfer approach in manipulating other addictive behaviors in rats suggests that the same approach will be useful in identifying the role of specific gene products in alcohol related behaviors as well.

Finally, viral vectors have been used in addition to knockout and transgenic techniques to analyze local protein expression as neurons grew and regenerated after injury (Holtmaat et al. 1998; Brooks et al. 1997). Although these studies were not specifically alcohol related, an improved understanding of neural regeneration at the molecular level can help answer questions concerning the extent to which cognitive impairment may be reversible following cessation of long-term drinking.

## Looking Forward

As the techniques described in this article continue to undergo improvement, they are likely to play an increasing role in alcohol research. Meanwhile, the concept of expression profiling is becoming increasingly practical. The simultaneous analysis of hundreds or thousands of genes from high-density micorarrays may enable researchers to monitor the expression status of cells and tissues in an entire organism or even across a population (van Ommen et al. 1999). Expression profiling could also become a powerful diagnostic tool, with each disease state being characterized by a typical gene expression pattern, or “signature”(van Ommen et al. 1999). Once these signatures are well described, they can be reduced to their basic patterns, which can help in differential diagnosis or help define stages of progression in a disease (van Ommen et al. 1999).

Detecting gene expression signatures could assist the development of individualized treatment strategies for alcoholism, while improving efforts to design targeted prevention programs to persons at high risk (van Ommen et al. 1999).
